# "Is Cybermedicine Killing You?" - University College London (UCL) Media Strategy Explained

**DOI:** 10.2196/jmir.7.4.e43

**Published:** 2005-07-28

**Authors:** Dominique Fourniol

Eysenbach and Kummervold [1] and Rada [2] criticize the handling of the Cochrane review retraction from a media perspective, and I wish to provide the following additional clarification of the actions of the UCL media relations office in this instance.

Had the withdrawal of the review been a permanent retraction with no further action to be taken by the authors or journal, the UCL media relations office would have issued a statement to this effect at the time via our media mailing lists.

Given that the review was withdrawn with a view to revision and republication at the earliest opportunity and not to permanent retraction, we decided to issue an updated press release once that process was complete. The release will both explain the error and provide the correct interpretation of the review, which will enable journalists to compare the original and revised papers and report on both the errors and the new, correct findings of the review. The release, anticipated later this year, will be sent to all journalists and websites which received the original October 2004 release.
